# Trueness and precision of digital implant impressions by intraoral scanners: a literature review

**DOI:** 10.1186/s40729-021-00352-9

**Published:** 2021-07-27

**Authors:** Minoru Sanda, Keita Miyoshi, Kazuyoshi Baba

**Affiliations:** Department of Prosthodontics, Showa University, 2-1-1 Kitasenzoku, Ota-ku, Tokyo, 145-8515 Japan

**Keywords:** Intraoral scanner, Dental implants, Impression accuracy

## Abstract

**Background:**

With the development of intraoral scanners, their trueness and precision have been evaluated in various studies. Through these studies, the amount of accuracy that can be expected from intraoral scanners has gradually been disclosed, at the same time, it was difficult to integrate the results of individual studies due to differences in evaluation methods between studies. The purpose of this article was to review the currently available evidence, summarise what is currently known about IOS, analyse the evaluation methods of each study, and list points to note when interpreting the results.

**Main text:**

Most of the studies were conducted in vitro. The accuracy is evaluated in situations such as single missing teeth, partially edentulous ridges with multiple missing teeth, and fully edentulous jaws. To evaluate the accuracy, direct measurement of distance or angle by coordinate measuring machines and calculation of surface deviation by superimposing surface data were predominantly performed. The influence of parameters such as the number of implants, distance between implants, angle between implants, and experience of the operator was evaluated. Many studies have shown that trueness tends to decrease as the distance between the implants and the scan range increases. It was agreed that the implant angle did not affect either trueness or precision. Regarding other factors, the results varied among studies. Therefore, the effects of these parameters are not clear.

**Conclusions:**

Heterogeneity in the research methodology was prevalent among the studies considered in this review. Therefore, we cannot make a decisive statement regarding the trueness and precision of digital implant impressions by IOSs. So far, the comparison of the numerical values of error between studies has yet to elucidate any clear answers, despite small methodological differences.

## Background

One of the most significant developments in dentistry during this century was the introduction of digital technology into dental treatment, denoted as digital dentistry. Digital impressions made with intraoral optical scanners (IOSs) have played a significant role in the facilitation of digital dentistry, dramatically changing the workflow of prosthetic treatment [1]. The advantages of digital impression techniques have already been well documented in several studies, with reports on simple data communication and storage [2], comfort for patients during the impression-making procedure [3], and options for an immediate evaluation of tooth preparations. Conventional impression procedures that use silicone impression materials and stone models are prone to dimensional changes, often because silicone impression materials shrink as a result of ongoing chemical reactions. Dental stones also expand owing to secondary reactions during setting. However, direct digital scanning of teeth is theoretically not associated with such changes. Consequently, digital impressions are expected to be more accurate than conventional impression methods, as demonstrated in several studies [4]. However, the use of this technique remains controversial. This is because the impression accuracy is affected by a variety of factors, such as the condition under which the impression is made [5].

In implant-supported prostheses, especially in screw-retained cases, a high impression accuracy is required because the passive fit of prostheses for implant platforms is crucial for the long-term stability of patients’ clinical outcomes [6]. Therefore, many studies have evaluated the accuracy of digital implant impressions using intraoral scanners under a variety of conditions.

This article aimed to review the accuracy of digital implant impressions by IOSs that have been published and then summarise the results. Additionally, any methodological issues of note will be mentioned, particularly when reviewing the literature regarding the accuracy of IOSs; otherwise, the results of the studies may be misinterpreted.

## Terminology

**Fig. 1:**
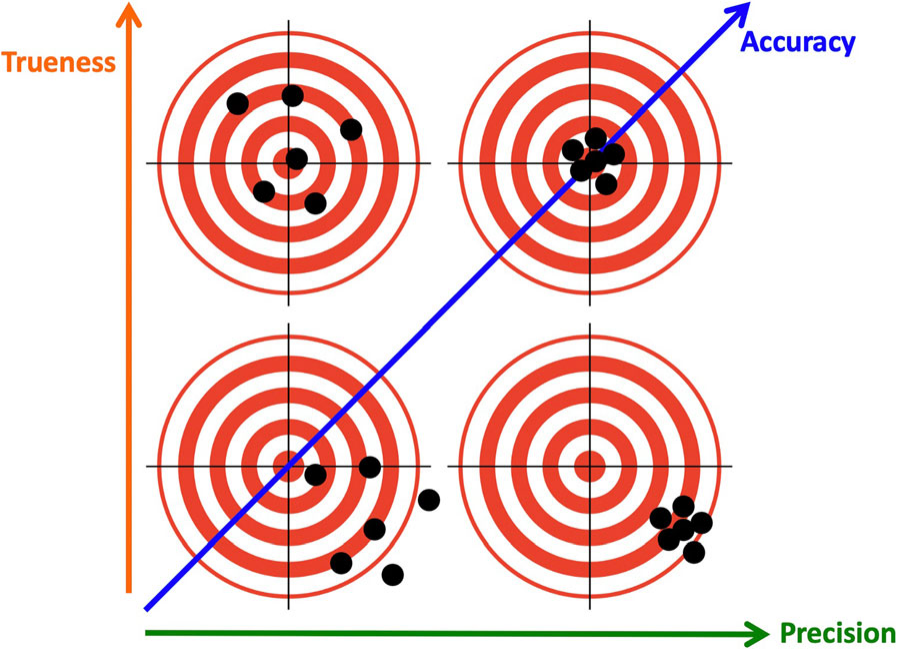
Conceptual image of the relationship between trueness, precision, and accuracy, as defined by ISO (1994). The centre of the target represents the “true value” provided by the reference data. The black dots represent test data obtained by repeated measurements. ISO, International Standard Organization

When discussing impression accuracy, the terms “accuracy”, “trueness”, and “precision” should be distinguished from each other. According to the definition by the International Standard Organization (ISO) in 1994, “accuracy” indicates the combination of “trueness” and “precision” [7], where “trueness” is defined as the “closeness of agreement between the arithmetic mean of a large number of test results and the true or accepted reference value” Meanwhile, “precision” was defined as “the closeness of agreement between different test results” (Fig. 1). Although “accuracy” is used as a synonym for “trueness” in some studies [8–10], this review follows the above-mentioned definition by the ISO.

## Methodological issues in evaluating trueness and precision

### Establishment of gold standard data

In order to evaluate “trueness”, the gold standard data to be used as the “true value” needs to be identified by the methods listed below.

#### Coordinate measuring machine

A coordinate measuring machine (CMM) is a device used to measure the geometry of an object. It has been used as a benchmark for accuracy in measuring solid objects for over five decades in the industrial field. Therefore, CMMs have been utilised in many studies to evaluate the accuracy of digital impression data [10–20]. CMMs typically specify a probe's position in terms of its displacement from a reference position in a three-dimensional Cartesian coordinate system (i.e., with XYZ axes). Various types of probes are used in CMMs, including mechanical, optical, laser, and white light.

The disadvantages of a CMM are that it lacks scan speed, and the number of points acquired from the model surface is limited when compared to industrial 3D scanners. Additionally, to acquire a precise measurement of a complicated shape using CMM, surface shape information is necessary before scanning can be performed. In addition, a CMM with a mechanical probe cannot detect small morphological structures such as fissure lines and gingival margins because the tip of the tactile probe has a certain diameter that limits its sensitivity.

#### Industrial 3D scanner

Industrial 3D scanners have been introduced in the industry over the last two decades. Data scanned with industrial 3D scanners are reported to be sufficiently accurate for use as a reference [21]. The size of the machine is smaller and costs less than the CMMs. Unlike CMMs, industrial 3D scanners can capture millions of points on an object’s surface simultaneously, even if the shape of the surface is complex. Currently available industrial 3D scanners display maximum deviations within a few micrometres [8].

#### Dental laboratory scanner

Several studies have used dental laboratory scanners instead of industrial 3D scanners to acquire reference data [9, 22]. Dental laboratory scanners are utilised to scan cast models produced from a conventional impression and create surface 3D data, which are then exported to CAD software to design the restorations. As listed in Table 1, the accuracy of industrial scanners ranges from 1 to 10 μm, whereas a laboratory scanner’s accuracy ranges from 2 to 10 μm, suggesting that the accuracy of digital impressions obtained by dental laboratory scanners is comparable to that of the industrial 3D scanner [14].

**Table 1:** Types of dental laboratory scanners, industrial 3D scanners, and CMMs used in the cited references and their accuracy

Accuracy of reference scanners			
Scanner	Manufacturer	Reference	Accuracy
Dental Laboratory Scanner			
Activity 880 scanner	Smart Optics	Amin 2016 [23] Marghalani 2018 [9]	Precision: 10 μm
D-250(3Shape)	3 Shape	Flugge 2016 [15]	2 μm
Freedom UHD®	DOF	Mangano 2019 [24]	5 μm
Iscan D103I	Imetric	Papaspiridacos 2016	6 μm
Iscan D104I	Imetric	Mangano 2016 [25]	Trueness < 5 μm, Precision < 10 μm
Lava Scan ST scanner	3M	Andriessen 2014 [20] Lee 2015	Not found
Industrial 3D scanner			
ATOS Compact Scan 5M	GOM	Arcuri 2019 [26]	
ATOS Core 80	GOM	Alikhasi 2018 [19] Nedelcu 2018 [8]	Precision 4 μm (Alikhasi 2018) [19] Precision 0.6 μm (Nedelcu 2018) [8]
Infinite Focus Standard	Alicona Imaging	Ender 2013	Trueness 5.3 ± 1.1 μm, Precision of 1.6 ± 0.6 μm (Ender 2013) Manufacturer’s information: Trueness 0.5 μm, Precision 0.1 μm
ScanRider	V-GER	Imburgia 2017 [27]	Trueness 5–10 μm, Precision 15–30 μm
stereoSCAN neo	AICON 3D Systems	KimRJY 2019 [28]	
Coordinate measuring machine (CMM)			Maximum error (*L* = Length: mm)
CONTURA	Zeiss	Kim 2019	Trueness: 1.5 + L/350 (Manufacturer’s information: Truness 0.7 μm, Precision 0.55 μm (Kim 2019)
Crista-Apex	Mitutoyo	Gimenez 2015 [18] Gimenez 2017 Gintaute 2018 [29]	Trueness: 1.9 + 3L/1000 μm
Crista Apex S	Mitutoyo	Menini 2017	Trueness: 1.9 + 3L/1000 μm
DEA Mistral	Brown & Sharpe	Alikhasi 2018 [19]	Trueness: 3.5 + L/250 μm
Edge ScanArm HD	FARO	Sami 2020 [30]	Trueness: 25 μm, Precision: 25 μm
Global Silver Performance 7.10.7	Brown & Sharpe	Chia 2017 [31] Tan 2019 [10]	Trueness: 1.9–2.0 μm
Leitz PMM 12106	Zeiss	Van der Meer 2012 [13]	Trueness: 0.3 μm, Precision: 0.1 μm
SmartScope Flash, CNC 300	Optical Gaging Products	Di fore 2019	Trueness: 2.8 + 5 L/1000 μm
UPMC 550-CARAT	Zeiss	Ajioka 2016 [14] Fukazawa 2017 [12]	Trueness: 0.8 + L/600 μm

The industrial 3D scanners and laboratory scanners used in the studies are listed in Table 1, along with their trueness and precision.

### Data acquisition and evaluation

For the evaluation of trueness and precision, the parameters to be compared must be determined and calculated, a process also known as data reduction. To evaluate their trueness, some studies compared the given distance and angulation measured by the IOS to those acquired by the gold standard method. In other studies, the 3D surface image data captured by the IOS and the gold standard method were superimposed, and their discrepancies were then calculated. To evaluate their precision, these parameters were compared between repeated measurements by IOSs.

#### Measurement of distance and angle error

**Fig. 2:**
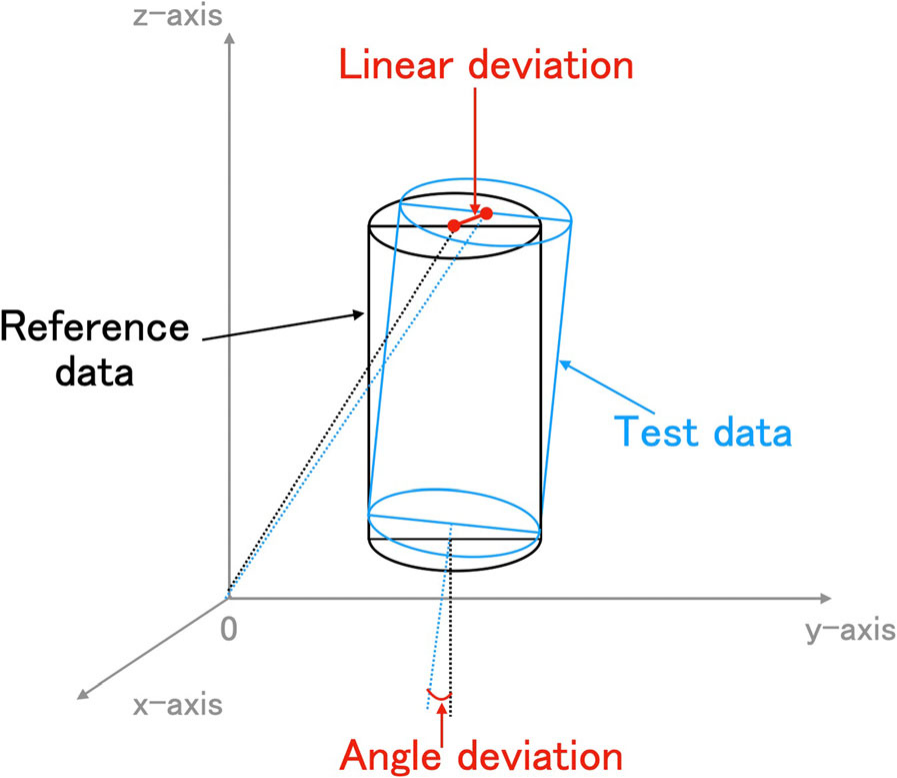
Schematic image: linear and angle errors using three-dimensional shift of test data from reference data

The linear error (or distortion) was measured as the deviation of certain positions between the reference and test data. The angle error was calculated by comparing the long axis angle of each scan body of the test data and the reference data with respect to the *XYZ* coordinate axes (Fig. 2).

**Fig. 3:**
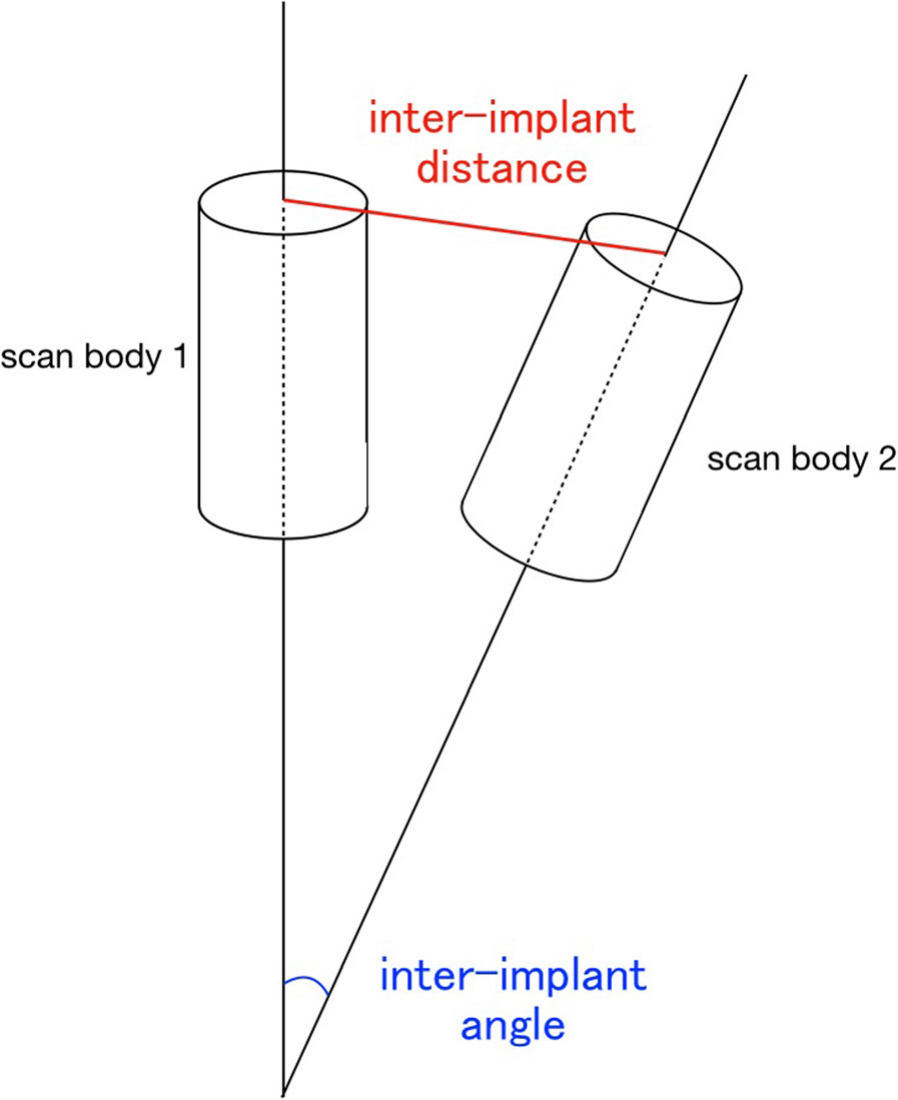
Schematic image indicating the inter-implant distance and inter-implant angle

To determine the inter-implant distances, the midpoints on the upper surface of the scan body were measured (Fig. 3). For the inter-implant angle, the angle between the long axes of the scan body was measured (Fig. 3).

The discrepancy in the inter-implant distances and inter-implant angles between the reference data and test data are termed the inter-implant distance error and inter-implant angle error, respectively.

#### Superimposition of surface data

To find discrepancies using a method other than distance and angle measurements, the STL data obtained by the IOS were superimposed on the reference data obtained with an industrial 3D scanner, laboratory scanner, or optical CMM in order to evaluate the discrepancy between the m[19].

The superimposition of the digital surface data is implemented using a “best-fit algorithm” [9, 22, 23, 25, 27, 32]. A best-fit algorithm is a method of alignment that causes a set of measured points or a set of actual feature centroids to match, as closely as possible, to that of their counterpart. The least-squares algorithm of the best-fit algorithm aligns the two-point sets by transforming one of the sets such that the sum of the squared distances between matching points in the two sets is minimal.

The advantage of the best-fit algorithm is that it can automatically calculate discrepancies between images. In addition, it is easy to intuitively understand the results by visualising the discrepancies between the images by colour.

**Fig. 4:**
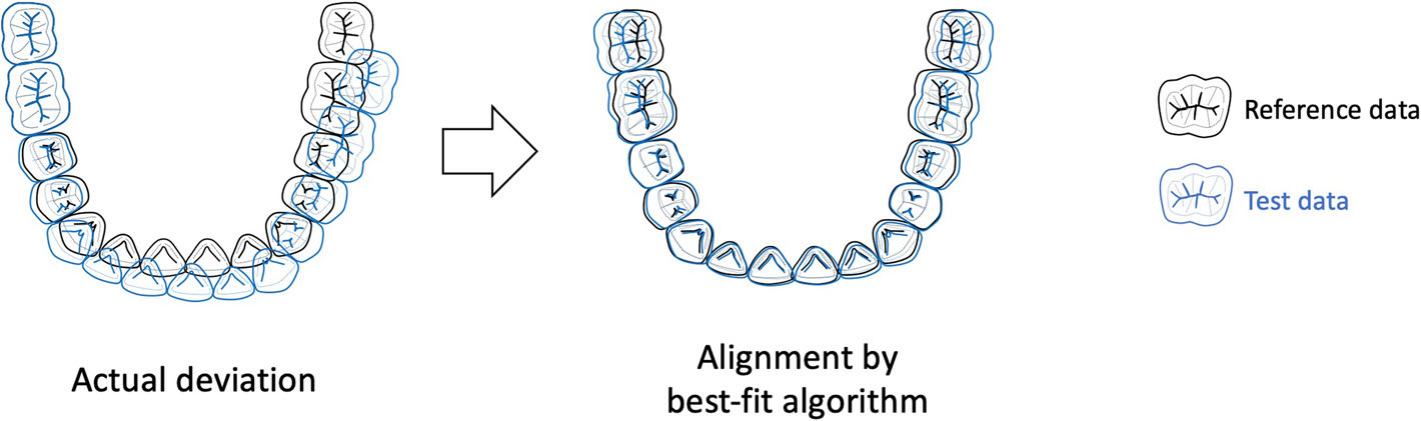
Schematic image showing positional shift of test data towards reference data through a best-fit algorithm. Due to its calculation methodology, the best-fit algorithm aligns test data with the reference data as closely as possible to its theoretical counterpart. Therefore, the actual positional relationship between the reference data and test data may deviate much more, and the deviation between the images may be underestimated

The disadvantage of the best-fit algorithm is that the deviation calculated using the best-fit algorithm may not be identical to the actual deviation that occurs during the scan. Owing to its calculation methodology, the best-fit algorithm aligns the test data with the reference data as closely as possible to its theoretical counterpart. Therefore, the actual positional relationship between the reference data and test data may deviate significantly, and the deviation between the images may be underestimated (Fig. 4). For scans right up to one quadrant, the best-fit algorithm seems to be suitable because the error caused by the superimposition itself between the test and reference data is within an acceptable range [33, 34]. However, the larger and more different the data, the greater the influence of the error, owing to the superimposition process [35].

#### Calculation of the magnitude of the error

When measuring using CMM, the three-dimensional position is defined by the values of the *x*-, *y*-, and *z*-axes of the Cartesian system. Therefore, the three-dimensional linear error and angle error require mathematical integration of the *x*, *y*, and *z* values. In terms of linear error, the integration method differed for each study. For example, some studies use the root sum square (RSS) formula, formulated as √(*x*^2^ + *y*^2^ + *z*^2^) [11, 12, 14, 16, 19, 31, 36, 37], while others use different formulas, such as the root mean square (RMS) √((*x*^2^ + *y*^2^ + *z*^2^)/3) [9, 22, 23, 28, 30, 38] [26, 30]. Hence, the difference in the calculation methods should be noted when interpreting these results.

Some suggest that the measurements should not be broken down into *x*-, *y*-, and *z*-components; rather, they should be directly carried out using engineering software. This is because the coordinate system defined for the measured data is not identical to the true coordinate system [13]. Therefore, different models can only be registered in a virtual common coordinate system. As the registration is based on the surface of the models and as these show minor errors, the positions of the model differ slightly. This introduces an error in their relative positions, making it unreliable to compare measurements broken down into *x*-, *y*-, and *z*-components.

Studies that used RSS, RMS, or other specific formulas are listed in Tables 2, 3, 4, 5, and 6.

**Table 2:** Summary of studies evaluated trueness of IOSs by CMM with CI. *CI*, conventional impression; *DI*, digital impression; *IOS*, intraoral scanner; *RMS*, root mean square √((*x*^2^ + *y*^2^ + *z*^2^)/3); *RSS*, root sum square ( √(*x*^2^ + *y*^2^ + *z*^2^))

Authors	Scanner for test data	Equipment for reference data	Conventional impression	Evaluated parameters as representative of accuracy	Operator	Models	Results about trueness		Conclusion
Ajioka et al. 2016 [14]	COS	UPMC 550-CARAT (CMM)	Open tray, non-splinted	IIDD & IIAD	1 experienced	Partially edentulous mandible with 2 implants (#35,36)	IIDD between implant #35 and #36 Calculation: RSS DI: 64.5 ± 19.0 μm CI: 22.5 ± 12.4 μm	5-mm height abutment COS: 0.42 ± 0.18° CI: 0.14 ± 0.01° 7-mm height abutment COS: 0.20 ± 0.20° CI: 0.15 ± 0.12°	Longer abutment reduces angle error in DI.
Chia et al. 2017 [31]	TRIOS	Global Silver Performance 7.10.7 (CMM)	Open tray	Linear and angle deviation	Not mentioned	Partially edentulous mandible with 2 implants (#44, #46) 0, 10, and 20° of buccolingual inter-implant angulation	Linear error in distance from reference point Calculation: RSS DI: 0°: 31 ± 14.2 μm 10°: 45 ± 3.4 μm 20°: 42 ± 9.9 μm CI: 0°: 18 ± 8.4 μm 10°: 33 ± 15.8 μm 20°: 36 ± 6.5 μm	Angule error towards *x*-axis in each inter-implant angle; DI: 0°: 0.041 ± 0.032° 10°: 0.55 ± 0.27° 20°: 0.80 ± 0.27° CI: 0°: 0.07 ± 0.06° 10°: 0.28 ± 0.30° 20°: 0.55 ± 0.06° Angule error towards *y*-axis in each inter-implant angle; DI: 0°: 0.10 ± 0.07° 10°: 0.11 ± 0.06° 20°: 0.08 ± 0.06° CI: 0°: 0.20 ± 0.13° 10°: 0.11 ± 0.08° 20°: 0.17 ± 0.13°	Bigger inter-implant angles tend to cause linear and angle error strain.
Alikhasi et al. 2018 [19]	TRIOS	DEA Mistral (CMM) ATOS Core 80 (CMM)	Open tray, non-splinted closed tray	IIDD & IIAD	1 experienced	Fully edentulous maxilla with 4 implants (#13, #15, #23, #25) #15, #25: distally 45° tilted	Calculation: RSS DI + internal connection; straight implant:188 ± 134 μm,/tilted implant:162 ± 103 μm DI+external connection; straight implant:195 ± 158 μm/tilted implant:165 ± 134 μm CI (open tray) + internal connection; straight implant: 280 ± 142 μm/tilted implant:389 ± 228 μm CI (open tray) + external connection; straight implant:711 ± 286 μm/tilted implant:364 ± 231 μm CI (closed tray)+internal connection; straight implant:885 ± 389 μm/tilted implant:721 ± 384 μm CI (closed tray)+external connection; straight implant: 797 ± 351 μm/tilted implant:442 ± 226 μm	Angle errors in each impression method and implant connection type: DI+internal connection; straight implant: 0.59 ± 0.72°/tilted implant:0.36 ± 0.37° DI + external connection; straight implant: 0.59 ± 0.72°/tilted implant:0.37 ± 0.38° CI (open tray) +internal connection; straight implant: 2.29 ± 1.33°/tilted implant: 4.77 ± 2.20° CI (open tray)+external connection; straight implant: 1.00 ± 0.45°/tilted implant:1.10 ± 0.39° CI (closed tray)+internal connection; straight implant: 4.10 ± 2.73°/tilted implant: 9.37 ± 6.90° CI (closed tray)+external connection; straight implant: 4.85 ± 1.46°/tilted implant:2.06 ± 0.97°	Trueness of impression: DI > CI with open-tray > CI with closed tray Connection type and implant angulation did not affect the trueness in DI.
Menini et al. 2018 [39]	TDS	Crista Apex S (CMM)	Open tray, non-splinted Open tray, splinted Closed tray	IIDD	3 experienced	Fully edentulous jaw with 4 implants	DI: 15 ± 11 to 19 ± 15 μm CI: 22 ± 23 to 63 ± 59 μm		DI showed better trueness than CI.
Tan et al. 2019 [10]	TDS TRIOS Ceramill Map400 (lab scanner) inEos X5 (lab scanner) D900 (lab scanner)		Open tray, Splinted	Linear and angle deviation	Not mentioned	Fully edentulous maxilla with 6 implants (#12, #14, #16, #22, #24, #26) 20 mm inter-implant distance	Calculation: RSS TDS: − 267 ± 85.4 to − 709 ± 66.8 μm TRIOS: 13.3 ± 47.4 to 166.8 ± 78.0 μm Ceramill Map400: − 4.8 ± 11.6 to 35.8 ± 31.9 μm inEos X5: 11.1 ± 9.3 to 45.4 ± 20.2 μm D900: 2.7 ± 32.6 to − 59.8 ± 40.0 μm	Angle deviation towards *x*-axis; TDS: − 0.06 ± 0.41 to − 2.25 ± 1.10° TRIOS: 0.02 ± 0.21 to − 2.19 ± 0.45° Ceramill Map400: − .04 ± 0.31 to − 2.35 ± 0.17° inEos X5: 0.05 ± 0.04 to − 1.62 ± 0.54° D900: − .15 ± 0.18 to − 1.94 ± 0.67° Angle deviation towards y-axis; TDS: − .14 ± 0.25 to 0.47 ± 0.32° TRIOS: 0.11 ± 0.20 to − .93 ± 0.29° Ceramill Map400: 0.03 ± 0.27 to − .74 ± 0.18° inEos X5: − .00 ± 0.16 to − .54 ± 0.16° D900: 0.22 ± 0.35 to 0.47 ± 0.36°	Shorter inter-implant distance reduce linear error in DI. TDS showed the poorest trueness for all linear errors in both models.
						Fully edentulous maxilla with 8 implants (#11, #13, #15, #17, #21, #23, #25, #27) 13-mm inter-implant distance	Calculation: RSS TDS: − 151.1 ± 32.8 to − 602.5 ± 70.0 μm TRIOS: − 9.1 ± 28.9 to 69.8 ± 109.2 μm Ceramill Map400: 9.8 ± 15.7 to 50.2 ± 20.9 μm inEos X5: 14.6 ± 7.5 to 66.4 ± 5.9 μm D900: − 4.2 ± 26.3 to − 34.7 ± 28.8 μm	Angle error towards x-axis; TDS: 0.02 ± 0.16 to − .69 ± 0.57° TRIOS: − .11 ± 0.30 to 0.53 ± 0.33° Ceramill Map400: 0.07 ± 0.14 to 0.60 ± 0.14° inEos X5: − .03 ± 0.07 to − .35 ± 0.11° D900: 0.04 ± 0.12 to − .31 ± 0.32° Angle error towards *y*-axis; TDS: 0.08 ± 0.15 to − 1.05 ± 0.30° TRIOS: 0.15 ± 0.19 to − .81 ± 0.33° Ceramill Map400: 0.28 ± 0.24 to − .84 ± 0.30° inEos X5: 0.02 ± 0.05 to − .88 ± 0.38° D900: 0.04 ± 0.11 to − .71 ± 0.24°	
Gintaute et al. 2018 [29]	TDS	Crysta-Apex (CMM)	Open tray, non-splinted	IIDD & IIAD	Not mentioned	Fully edentulous mandible with 2 straight implants (#32, #42)	18.5 ± 19.8 μm	0.04 ± 0.05°	DI and CI are comparable but DI can be applied for fully edentulous jaw with multiple implant cases. Although some statistically significant differences in errors between the impression methods and models, they were within clinically acceptable range.
						Fully edentulous mandible with 4 straight implants (#32, #34, #42, #44)	9.5 ± 16.0 μm	0.17 ± 0.14°	
						Fully edentulous mandible with 2 straight implants (#32, #42) and 2 distally angulated implants (#34, #44)	35.8 ± 24.2 μm	0.22 ± 0.19°	
						Fully edentulous mandible with 6 straight implants (#32, #34,#36, #42, #44, #46)	31.1 ± 27.1 μm	0.24 ± 0.22°

**Table 3:** Summary of studies evaluated trueness of IOSs by CMM without CI. CI: conventional impression; DI: digital impression; IOS: intraoral scanner; RMS: root mean square √((*x*^2^ + *y*^2^ + z2)/3); RSS: root sum square ( √(*x*^2^ + *y*^2^ + *z*^2^))

Authors	Scanners for test data	Equipment for reference data	Evaluated parameters as representative of accuracy	Operator	Models	Results about trueness		Conclusion
Giménez B et al. 2015	COS	Crista Apex (CMM)	IIDD	2 experienced, 2 inexperienced	Fully edentulous maxilla with 6 implants (#12, #15, #17, #22, #25, #27) #15 distally 30° tilted #25 mesially 30° tilted	Angle effect on IIDD: Angulated implants: − 20.2 ± 21.9 μm non-angulated implants: − 37.9 ± 26.2 μm Operator’s effect on IIDD: ﻿Experienced operator: − 30.8 ± 25.9 μm Inexperienced operator: 13.3 ± 51.2 μm Implant depth effect on IIDD: Deep implants: − 34.3 ± 18.7 μm Normal-depth implants: − 28.3 ± 29.8 μm		﻿Angle and depth of implants did not affect trueness.Experience of operator affected on trueness.
Fukazawa et al. 2017 [12]	COS TDS2 TDS3 TRIOS KaVo(lab scanner)	UPMC 550-CARAT (CMM)	IIDD	1 experienced	Partially edentulous mandible with 2 implants (#35, #36)	Rate of changes in IIDD (calculation: RSS) COS: 0.30 % TDS2:0.17 % TDS3: 0.17 % TRIOS: 0.05 % Kavo: 0.02 %		IOSs are useful for implant therapy for multiple missing teeth.
					Partially edentulous mandible with 2 implants (#45, #47)	COS: 0.45 % TDS2: 0.38 % TDS3: 0.32 % TRIOS: 0.11 % KaVo: 0.07 %		
Gimenez et al. 2017 [40]	TDS	Crista Apex (CMM)	IIDD & IIAD	2 experienced, 2 inexperienced	Fully edentulous maxilla with 6 implants (#12, #15, #17, #22, #25, #27) #15: mesially 30° tilted #12: 4 mm subgingiva #22: 2 mm subgingiva #25: distally 30° tilted	Mean IIDD;5.38 ± 12.61 μm to − 26.97 ± 50.56 μm Mean linear errors in 1st and 2nd quadrant; 1st quadrant: 7.6 ± 17.6 μm 2nd quadrant: − 10.3±39.2 μm	Mean IIAD: 0.16 ± 0.04 to − 0.43 ± 0.1° Mean angle errors in 1st and 2nd quadrant; 1st quadrant: 0.21 ± 0.17° 2nd quadrant: 0.28 ± 0.16°	TDS achieves acceptable trueness. Scan body visibility, experience of operator and scan range affects trueness of DI.
Di Fore et al. 2019	TDS Omnicam 3D progress CS3500 CS3600 Emelard Dental wings	SmartScope Flash,CNC 300 (CMM)	Linear deviation	1 experienced	Fully edentulous mandible with 6 implants (#32, #34, #36, #42, #44, #46)	Mean linear deviations; TDS: 31 ± 8 μm TRIOS: 32 ± 5 μm Omnicam: 71 ± 55 μm CS3600: 61 ± 14 μm CS3500: 107 ± 28 μm Emelard: 101 ± 38 μm 3D progress: 344 ± 121 μm Dental Wings: 148 ± 64 μm		Some scanners are not suitable for DI in full-arch implant-supported fixed dental prosthesis.
Sami et al. 2020 [30]	TDS TRIOS Omnicam Emerald	Edge ScanArm HD (CMM)	Linear deviation	1 experienced	Fully edentulous mandible with 6 implants (5mm apart each)	Calculation: Arithmetic average (95% CI) TDS: − .02mm [− .09, 0.78] TRIOS: − .13mm [− .21, 0.86] Omnicam: − .13mm [− .22, − .04] Emerald: − .05mm [− .09, − .02] Calculation: RMS [95% CI] TDS: 0.70mm [0.62, 0.78] TRIOS: 0.74mm [0.62, 0.86] Omnicam: 0.75mm [0.70, 0.79] Emerald: 0.81mm 0.77, 0.85]		No difference among IOSs.

**Table 4:** Summary of studies evaluated trueness of IOSs by industrial 3D scanner. *CI*, conventional impression; *DI*, digital impression; *IOS*, intraoral scanner; *RMS*, root mean square √((*x*^2^ + *y*^2^ + *z*^2^)/3), *RSS*: root sum square ( √(*x*^2^ + *y*^2^ + *z*^2^))

Authors	Scanners for test data	Equipment for reference data	Conventional Impression	Evaluated parameters as representative of accuracy	Models	Results about trueness		Conclusion
Amin et al. 2016 [23]	Omnicam TDS	Activity 880 scanner (lab scanner)	Open tray, Splinted	Linear deviation	Fully edentulous mandible with 5 implants (at midline, #33, #35, #43, #45) #35: distally 10° tilted #45: distally 15° tilted	Mean linear deviation (calculation: RMS);TDS: 19.3 ± 2.8 μm Omnicam: 46.4 ± 7.3 μm CI: 167.9 ± 50.4 μm		Trueness order: TDS, Omnicam > CI with splinted open-tray\TDS > Omnicam
Imburgia et al. 2017 [27]	CS3600 Trios3 Omnicam TDS	ScanRider (industrial scanner)	No CI	Linear deviation	Partially edentulous maxilla with 3 implants (#23, #24, #26)	CS3600 (45.8 ± 1.6 μm) > TRIOS3 (50.2 ± 1.6 μm) > Omnicam (58.8 ± 1.6 μm) = TDS (61.4 ± 3.0 μm)		Difference of IOS affected trueness Partially edentulous model showed better trueness than fully edentulous model in all IOS.
					Fully edentulous maxilla with 6 implants (#11, #14, #16, #21, #24, #26)	CS3600(60.6 ± 11.7 μm) > TDS (106.4 ± 23.1 μm) TRIOS3 (67.2 ± 6.9 μm) > TDS (106.4 ± 23.1 μm) Omnicam (66.4 ± 3.9 μm) > TDS (106.4 ± 23.1 μm)		
Van der Meer et al. 2012 [13]	Bluecam iTero COS	Leitz PMM 12106 (CMM)	No CI	IIDD & IIAD	Partially edentulous mandible with 3 implants (#36, #41, #46)	IIIDD; #36-41 COS: 14.6 ± 12.7 μm iTero: 70.5 ± 56.3 μm Bluecam:79.6 ± 77.1 μm #36–46 COS: 23.5 ± 14.2 μm iTero: 61.1 ± 53.9 μm Bluecam: 81.6 ± 52.5 μm	IIAD; #36–41 COS: 0.21 ± 0.04° iTero: 0.35 ± 0.34° Bluecam: 0.63 ± 0.55° #36–46 COS: 0.47 ± 0.14° iTero: 0.42 ± 0.17° Bluecam: 0.44 ± 0.32°	Increase in linear and/or angle errors over the length of the arch were observed(not statistically significant).
Arcuri et al. 2019 [26]	Trios3	ATOS Compact Scan 5M (industrial scanner)	No CI	Linear deviation and angle deviation	Fully edentulous maxilla with 6 implants (#12, #14, #16, #22, #24, #26)	Calculation: ASS (|Δ*x*|+|Δ*y*|+|Δ*z*|) Mean linear deviation of each scan body’s material [95% CI] Titanium: 99.3 μm [78.3, 120.3] Peek: 54.7 μm [33.7, 75.7] Peek-titanium: 196 μm [175.4, 217.5] Mean linear error of each implants’ position #12: 100 μm #14: 126 μm #16: 117 μm #22: 81 μm #24: 104 μm #26: 172 μm	Scan body’s angle (deg) deviations Titanium: 0.71 ± 0.29° Peek: 0.64 ± 0.27° Peek-Titanium: 0.76 ± 0.36°	Implant angulation affected the linear deviationImplant position affected the angle deviation Materials of scan body affected accuracy of DI (PEEK was better than titanium and PEEK+titanium) Operator did not affect trueness
Kim RJY et al. 2019 [28]	Omnicam CS3600 i500 iTero Trios	stereoSCAN neo (industrial scanner)	No CI	Linear deviation	Partially edentulous mandible with 6 implants (#33,#35,#37,#43,#45,#47) #37: mesially 30° tilted #47: distally 30° tilted	Calculation: RMS Omnicam: 75.07 [25.97, 147.85] CS3600: 72.70 [30.50, 158.58] i500: 82.25 [38.20, 171.92] iTero: 68.52 [26.65, 155.05] TRIOS3: 90.26 [43.22, 218.02]		All the IOSs showed bigger linear deviation with an increasing distance. The direction and magnitude of deviation differed among jaw regions and IOSs. All the IOSs were similar for unilateral arch scanning, while i500 and TRIOS 3 showed better trueness than the others for partially edentulous model.

**Table 5:** Summary of studies evaluated trueness of IOSs by superimposing digital data. *CI*, conventional impression; *DI*, digital impression; *IOS* intraoral scanner; *RMS*, root mean square √((*x*^2^ + *y*^2^ + *z*^2^)/3); *RSS*, root sum square ( √(*x*^2^ + *y*^2^ + *z*^2^))

Authors	IOSs	Equipment for reference data	Conventional impression	Evaluated parameters as representative of accuracy	Models	Results about trueness	Conclusion
Papaspyridakos 2015 [37]	Trios	Iscan D103i (lab scanner)	Splinted, implant level Non-splinted, implant level Splinted, abutment level Non-splinted, abutment level	IIDD	Fully edentulous mandible with 5 implants(at midline, #33, #35, #43, #45) #35: distally 10° tilted #45: distally 15° tilted	Calculation: RSS, [95% CI] DI: 19.38 μm [11.54–26.21] CI (splinted, implant level): 7.42 μm [5.28–10.88] CI (non-splinted, implant level): 17.65 μm [13.19–76.49] CI (splinted, abutment level): 13.05 μm [10.46–23.67] CI (non-splinted, abutment level): 8.23 μm [4.01–12.13]	DI is as accurate as CI. The implant angulation up to 15° did not affect the trueness of DI.
Mangano et al. 2016 [25]	Trios2 CS3500 Zfx Intrascan Planscan	Iscan D104I (lab scanner)	No CIs	Surface deviation IIDD & IIAD	Partially edentulous maxilla with 3 implants (#21, #24, #26)	TRIOS2 (71.2 ± 19.5) > Planscan (233.4 ± 62.6) CS3500 (47.8 ± 7.3) > Zfx(117.0 ± 28.6), Planscan (233.4 ± 62.6) Zfx (117.0 ± 28.6) > Planscan (233.4 ± 62.6)	Edentulous type didn’t affect trueness. Difference of IOS affected trueness
					Fully edentulous maxilla with 6 implants (#11, #14, #16, #21, #24, #26)	TRIOS2 (71.6 ± 26.7 ) > Planscan(253.4 ± 13.6) CS3500 (63.2 ± 7.5) > Zfx (103.0 ± 26.9), Planscan(253.4 ± 13.6) Zfx (103.0 ± 26.9) > Planscan (253.4 ± 13.6)	
Mangano et al. 2019 [24]	TRIOS3 CS3600 Omnicam DWIO Emerald	Freedom UHD (lab scanner)	No CIs	Surface deviation	Partially edentulous maxilla with 3 implants (#14, #16, #23)	#14, #16: CS3600 (23 ± 1.1) > Trios3 (28.5 ± 0.5) > Omnicam (38.1 ± 8.8) > Emerald(49.3 ± 5.5) > DWIO(49.8 ±5) #23: CS3600 (15.2 ± 0.8) > Trios3 (22.3 ± 0.5> DWIO (27.8 ± 3.2) > Omnicam (28.4 ± 4.5) > Emerald (43.1 ± 11.5)	Trueness was different among IOSs. Linear deviation was bigger in larger edentulous space.
					Fully edentulous maxilla with 6 implants (#11, #14, #16, #21, #24, #26)	CS 3600 (44.9 ± 8.9) > Trios3 (46.3 ± 4.9) > Emerald (66.3 ± 5.6) > Omnicam (70.4 ± 11.9) > DWIO(92.1 ± 24.1)	
Roig et al. 2020 [38]	Omnicam TDS TRIOS3 CS3600	D810 (lab scanner)	Open tray, splinted open tray, non-splinted closed tray	IIDD & IIAD	Partially edentulous maxilla with 2 implants (#14, #16)	Calculation: RMS Omnicam: 0.225 mm, 0.063 mm TDS: 0.235 mm, 0.078 mm TRIOS3: 0.019 mm, 0.024 mm CS3600: 0.012 mm, 0.018 mm	TRIOS3 and CS3600 showed better trueness compared to that of closed tray, Omnicam, and TDS

**Table 6:** Summary of the results of researches evaluated IOS’s precision *CI*, conventional impression; *DI*, digital impression; *IOS*, intraoral scanner; *RMS*, root mean square √((*x*^2^ + *y*^2^ + *z*^2^)/3); *RSS*, root sum square ( √(*x*^2^ + *y*^2^ + *z*^2^))

Article	Scanners for test data	Equipment for reference data	Evaluated parameters as representative of accuracy	Models	Results about precision		Conclusion
Ajioka et al. 2016 [14]	COS	UPMC 550-CARAT (CMM)	IIDV & IIAV	Partially edentulous mandible with 2 implants (#35, 36)	Mean IIDV (Calculation: RSS); DI: 15.6 ± 10.9 μm CI: 13.5 ± 8.6 μm	Mean IIAV; 5-mm height abutment; DI: 0.42 ± 0.17° CI: 0.14 ± 0.06° 7-mm height abutment; DI: 0.19 ± 0.09° CI: 0.16 ± 0.09°	Longer abutment or scan body reduces angle error in DI.
Flügge et al. 2016 [15]	iTero TRIOS TDS	D250 (lab scanner)	IIDV	Partially edentulous mandible with 2 implants (#35, #36) Partially edentulous mandible with 5 implants (#37, #36, #34, #45, #47)	The results were provided by a graph without numerical data.		Longer inter-implant distance deteriorated precision in TDS, but not in iTero and TRIOS. The angle measurement didn’t show deterioration in precision for longer inter-implant distances in all IOSs.
Mangano et al. 2016 [25]	TRIOS2 CS3500 Zfx Intrascan Planscan	Iscan D104I (lab scanner)	Linear variation Angle variation Surface variation	Partially edentulous maxilla with 3 implants (#21, #24, #26)	TRIOS2 (51.0 ± 18.5 μm) = CS3500 (40.8 ± 6.4 μm) > Zfx (126.2 ± 21.2 μm) > Planscan (219.8 ± 59.1 μm)		Difference of IOS affected precision Edentulous type didn’t affect precision
				Fully edentulous maxilla with 6 implants (#11, #14, #16, #21, #24, #26)	TRIOS2 (67.0 ± 32.2 μm) = CS3500 (55.2 ± 10.4 μm) > Zfx (112.4 ± 22.6 μm) > Planscan (204.2 ± 22.7 μm)		
Imburgia et al. 2017 [27]	CS3600 TRIOS3 Omnicam TDS	ScanRider (industrial scanner)	Surface variation	Partially edentulous maxilla with 3 implants (#23, #24, #26)	Mean surface variation; TDS: 19.5 ± 3.1 μm TRIOS3: 24.5 ± 3.7 μm CS3600: 24.8 ± 4.6 μm Omnicam: 26.3 ± 1.5 μm No significant difference between IOSs		Difference of IOS didn't affect precision Edentulous type affected precision in some IOSs. Precision in partially edentulous models was better than fully edentulous models except TRIOS 3.
				Fully edentulous model with 6 implants (#11, #14, #16, #21, #24, #26)	Mean surface variation; TRIOS3: 31.5 ± 9.8 μm Omnicam: 57.2 ± 9.1 μm CS3600: 65.5 ± 16.7 μm TDS: 75.3 ± 43.8 μm No significant difference between IOSs		
Mangano et al. 2019 [24]	TRIOS3 CS3600 Omnicam DWIO Emerald	Freedom UHD (lab scanner)	Surface variation	Partially edentulous maxilla with 3 implants (#14, #16, #23)	Mean surface variation and order of precision in each edentulous site; #23 CS 3600 (11.3 ± 1.1 μm) > TRIOS3 (15.2 ± 0.8 μm) > DWIO (27.1 ± 10.7 μm) > Omnicam (30.6 ± 3.3 μm) > Emerald (32.8 ± 10.7 μm) #14, #16 CS 3600 (17 ± 2.3 μm) > TRIOS3 (21 ± 1.9 μm) > Emerald (29.9 ± 8.9 μm) > DWIO (34.8 ± 10.8 μm) > Omnicam (43.2 ± 9.4 μm)		Precision was different among IOSs. Linear error was bigger in larger edentulous space.
				Fully edentulous maxilla with 6 implants (#11, #14, #16, #21, #24, #26)	TRIOS3 (35.6 ± 3.4) > CS 3600(35.7 ± 4.3) > Emerald (61.5 ± 18.1) > Omnicam(89.3 ±14) > DWIO (111 ± 24.8)		
Di Fore et al. 2019	TDS Omnicam 3D progress CS3500 CS3600 Emelard Dental wings	SmartScope Flash, CNC 300 (CMM)	IIDV	Fully edentulous mandible with 6 implants (#32, #34, #36, #42, #44, #46)	The results were provided by a graph without numerical data.		Linear relationship between the errors and inter-implant distance was detected for TDS and CS3600. Some scanners are not suitable for DI in full-arch implant-supported fixed dental prosthesis.
Miyoshi et al. 2019 [41]	CS3600 TRIOS2 Omnicam TDS	3Shape D810 (lab scanner)	Surface variation	Fully edentulous maxilla with 6 implants (#12, #14, #16, #22, #24, #26)	Mean surface variation; I. With increase in the number of implants; TRIOS2: 28.6 ± 10.0 μm Omnicam: 18.7 ± 1.4 μm CS3600: 21.3 ± 6.1 μm TDS: 16.4 ± 5.3 μm Significant difference between IOSs II. Without increase in the number of implants; TRIOS2: 31.8 ± 5.6 μm Omnicam: 19.7 ± 6.3 μm CS3600: 21.6 ± 8.2 μm TDS: 16.2 ± 7.2 μm Significant difference between IOSs		Difference of IOS and impression range and their interactions were statistically significant. Precision of the IOSs were comparable with dental laboratory scanner in a limited range. Precision of IOS deteriorated as the ROI expanded
Roig et al. 2020 [38]	Omnicam TDS TRIOS3 CS3600	D810 (lab scanner)	Surface variation	Partially edentulous maxilla with 2 implants (#16 and #14)	Mean surface variation; Omnicam: 0.034mm TDS: 0.027 mm TRIOS3: 0.029 mm CS3600: 0.042 mm		DI showed better precision than CI.

## Literature review

### Search strategy

Online electronic databases, including the MEDLINE database and the Cochrane Central Register of Controlled Trials, were searched by a reviewer (MS) without any language filters for articles published between 2010 and 1 May 2020. The search terms included “intraoral scanner”, “accuracy”, “trueness”, “precision”, “digital impression”, “Dental Impression Technique” [MeSH] In addition, reference lists of relevant articles were manually searched to identify eligible studies. The two authors (MS and KM) independently screened the titles and abstracts of the retrieved articles to identify studies that fulfilled the predetermined eligibility criteria. They also reviewed the full texts of the shortlisted articles to arrive at the final selection of studies for inclusion in this narrative review. In addition, previous review articles on the subject were searched, as well as the reference lists of the articles already identified for further potentially relevant publications. Although there was no language restriction, the minimum requirement was access to an English version of the title and the abstract.

## Trueness evaluation

Due to the limited access of CMMs, industrial 3D scanners, or dental laboratory scanners into the oral cavity. It is generally impossible to establish reference data in real patients. Indeed, there is no in vivo study that has investigated the trueness of the digital impression for dental implant and all of the following reviewed in vitro studies are laboratory-based.

### Linear and angle error evaluation using CMM (Table 2)

#### Studies that evaluated digital impression compared to conventional methods

Gintaute et al. evaluated the trueness of digital impressions and conventional impressions using four types of reference models with different inter-implant distances and inter-implant angles: (1) two straight, (2) four straight, (3) two straight and two tilted, and (4) six straight dental implants [29]. The inter-implant distances and inter-implant angles of the reference models were measured as reference data using CMM. As test groups, digital impressions of the reference models that were acquired using TDS and STL data were analysed using 3D evaluation software. Polyether and vinyl polysiloxane impressions were utilised for the conventional impressions, and stone casts were made from the impressions and subsequently measured using CMM.

Regarding implant orientations (1), (3), and (4), digital impressions showed significantly lower inter-implant distance errors than conventional impressions. In terms of inter-implant angle error, the digital impressions exhibited significantly higher trueness than the conventional impression in all four reference models. However, the inter-implant distance and inter-implant angle errors were within 100 μm and 0.5°, respectively, which the authors judged to be clinically acceptable (Table 2).

Ajioka et al. evaluated the trueness of the digital impression by COS and the influence of the height of the abutments on the angle error [14]. A reference model with two implants in a partially edentulous model (#35 and #36) was prepared. Conventional models made of plaster were fabricated from a reference model using a silicone impression. For the distance measurements, two ball abutments were connected to the implants, and the distance between the centres of the balls of the abutments was measured. For the angle measurements, pairs of healing abutments that were 5 mm or 7 mm tall were connected, and the angulation between the healing abutments was measured. The reference model and conventional models were measured using CMM. The distance errors of the digital impressions were slightly greater than those of the conventional impressions. The angulation error was also greater for the 5-mm digital impressions but was not significantly different from the conventional method when 7-mm abutments were connected. Suggesting that a longer abutment or scan bodies may improve the trueness of digital impressions (Table 2).

Chia et al. evaluated the trueness of digital impressions for a three-unit bridge supported by two implants with three different inter-implant angles [31]. Three reference models with buccolingual inter-implant angulations of 0°, 10°, and 20° were fabricated. The scanned bodies connected to the reference models were scanned using IOS. The conventional impressions of each reference model were made using polyether impression materials, and conventional plaster models were fabricated. The reference and conventional models were measured using CMM. The impression technique (*p* = 0.012) and implant angulations (*p* = 0.007) had a significant effect on the linear error. In terms of the angle effect, the digital impression group showed consistent linear and angle errors, irrespective of inter-implant angulation. In addition, digital impressions tended to replicate the implant position more apically than the actual position (Table 2).

Menini et al. compared the trueness of digital impressions and conventional impressions using a full-arch edentulous reference model with four implants [39]. CMM was used to measure the implant angulation and inter-implant distances in the reference model as well as on the conventionally fabricated casts. Conventional impression data and digital impression data were compared with the reference data measured using the reference model. The trueness of the conventional group, as evaluated by the linear error, was inferior to that of the digital impression data (Table 2).

Tan et al. compared the trueness of digital impressions using two IOSs (Trios and TDS) to conventional impressions [10]. They used two reference models with edentulous maxillary arches with six or eight implants. The inter-implant distances were approximately 20 mm in the six implant models and 13 mm in the eight implant models. The centre positions at the implant platform level on the reference models were detected using the CMM. The results of this study showed that narrower inter-implant distances might decrease IOS linear errors. In addition, TDS showed a greater linear error than Trios (Table 2).

Alikhasi et al. investigated the trueness of digital impressions by Trios using two maxillary edentulous reference models with different internal or external implant connections, with two anterior straight and two posterior angulated implants [19]. Conventional plaster models were fabricated from silicone impressions using an open tray or closed tray. The conventional and reference models were measured using an optical CMM. STL datasets from the digital impression were superimposed on the reference data to assess the angle and linear errors. Digital impressions demonstrated superior outcomes compared to conventional methods. While the trueness of digital impressions was not affected by the type of connection and angulation, conventional impressions were significantly affected by these factors (Table 2).

### Studies that exclusively evaluated digital impressions

Giménez et al. conducted two studies evaluating the trueness of a digital impression by COS using a reference model with six implants (#27, #25, #22, #12, #15, and #17). The implant at #25 was mesially inclined by 30°, the implant at #15 was distally inclined by 30°, and the implants at #22 and #12 were placed 2 mm and 4 mm subgingivally, respectively [18, 40]. Two experienced and two inexperienced operators performed the scans. The CMM was used to measure the reference model, and the linear error was calculated. The angulation (*p* = .195) and depth of the implant (*p* = .399) measured by digital impression did not deviate significantly from the true values. Additionally, the experience of the operator significantly influenced the trueness of digital impressions (Table 3).

Sami et al. evaluated the trueness of digital impressions from four IOSs (TDS, TRIOS, Omnicam, and Emerald Scanner) [30]. An edentulous reference mandible model with six implants was fabricated and measured using four IOSs and an optical CMM. Data from the four IOSs were superimposed on the reference data, and the discrepancy between them was evaluated. The results indicated no statistical or clinical differences among the IOSs (Table 3).

Fukazawa et al. evaluated the trueness of the inter-implant distance on the surface data captured by several IOSs and a laboratory scanner and compared these to measurements acquired by CMM as references. They prepared two reference models with missing teeth at #35, #36, #45, #46, and #47. Model A had two neighbouring implant analogues at #35 and #36, whereas model B had implant analogues at #45 and #47. They found that the IOS error values were greater than the errors of the laboratory scanner. The linear error tended to be greater with longer inter-implant distances (model B) (Table 3).

Di Fiore et al. compared the trueness of the digital impression from 8 IOSs (TDS, Trios, Omnicam, 3D progress, CS3500, CS3600, Planmeca Emelard, and Dental Wings) in a full-arch implant-supported FPD [42]. An acrylic model of an edentulous mandible with six implants was used as the reference model. They evaluated the 3D position of the scan bodies and inter-implant distances captured by the IOSs in comparison to those captured by the CMM. The deviations of the 3D positions of the scan bodies were calculated using the best-fit algorithm. The distances between all combinations of the six scan bodies (15 pairs) were calculated from the STL data using analysis software and were compared to the reference data measured by CMM. The 3D position results of the implants, as measured by each IOS, showed that the TDS and Trios showed the best trueness among the IOSs, followed by Omnicam and CS3600 with average performance; CS3500 and Planmeca Emelard presented a middle-low performance, while the 3D progress and Dental Wings showed the lowest performance. The inter-implant distance analysis showed that shorter inter-implant distances corresponded to better trueness when using the True Definition and CS3600 devices (Table 3).

### Summary of the results of studies that utilised CMM for trueness evaluation

Except for one study, digital impressions showed superior trueness to conventional impressions. A longer inter-implant distance tended to deteriorate trueness. Three studies found a difference in trueness among manufacturers of IOS, while one study did not. The experience of operators in digital impressions positively affected the trueness of digital impressions. A longer scan body seemed to contribute to better trueness. The inter-implant angle and the difference in platform configuration (internal or external) did not affect the trueness of digital impressions.

### Linear and angle errors by industrial 3D scanners

#### Studies that evaluated digital impression compared to conventional methods

Amin et al. evaluated the trueness of digital impressions from two IOSs (Omnicam and TDS) using a full mandibular edentulous reference model with five implants [23]. The three median implants were parallel to each other. The far-left and far-right implants were inclined by 10° and 15° distally, respectively. A splinted open-tray technique was used for conventional polyether impressions to fabricate conventional models. The reference and conventional models were scanned using an industrial 3D scanner. The digital impression data from the reference model that was captured by the IOSs and the data from the conventional model captured by the industrial 3D scanner were superimposed with the reference data and evaluated using the best-fit algorithm. The full-arch digital impression using TDS and Omnicam showed significantly higher trueness than the conventional impressions using the splinted open-tray method (Table 4).

#### Studies that exclusively evaluated digital impression

Van der Meer et al. evaluated the trueness of three IOSs using dentate reference models with three implant analogues (#36, #41, #46) [13]. They measured the inter-implant distances and inter-implant angles of #36–41 and #36–46. An industrial 3D scanner and engineering software were used to obtain the reference data. The inter-implant distances and inter-implant angles captured by the IOSs were compared with the reference data, and the trueness of each scanner was evaluated. The distance discrepancies between the IOS data and reference data varied depending on the IOS and scanning range. An increase in distance and/or angle errors were associated with a larger scanning range but this trend was not statistically significant (Table 4).

Imburgia et al. compared the trueness of four IOSs (CS3600, Trios3, Omnicam, TDS) using a partially edentulous model with three implants and a fully edentulous model with six implants. The reference data were acquired using an industrial 3D scanner, which was superimposed with the scanned data from each IOS [27]. Trueness differed among IOSs. For all scanners, the trueness values obtained from the partially edentulous model were significantly better than those obtained from the fully edentulous model (Table 4).

Arcuri et al. evaluated the influence of implant scan body materials on digital impressions using an IOS (Trios3) [26]. An edentulous maxillary model with six internal connection implants was scanned using an industrial 3D scanner to acquire the reference data. Scanned bodies made of three different materials (polyetheretherketone (peek), titanium, and polyetheretherketone with a titanium base (peek-titanium)) were scanned by three operators using the IOS. These data were superimposed on the reference data using a best-fit algorithm. Linear and angle errors were assessed, and a significant influence of the type of material was identified (*p* < 0.0001), where the peak showed the best results in terms of both linear and angular measurements, followed by titanium and the peek-titanium (Table 4).

Kim et al. evaluated the trueness of digital impressions by five IOSs using a partially edentulous model [28]. A 3D printed partially edentulous mandible model made of Co-Cr, with six bilaterally positioned implants in the canine, second premolar, and second molar area served as the reference model. Reference data were acquired with an industrial 3D scanner, and the test data were obtained from five IOSs (Omnicam, CS3600, i500, iTero Element, and TRIOS3). For data from each IOS, the *XYZ* coordinates of the implants were obtained, and the deviations from the reference data were calculated.

The linear and angle errors differed depending on the implant position and the IOS. Regardless of the IOS type, the implants positioned on the left second molar, nearest to the scanning start point, showed the smallest linear error. The error generally increased further away from the scanning start point towards the right second molar (Table 4).

## Summary of results from studies that utilised an industrial 3D scanner for trueness evaluation

Results from the studies that used an industrial 3D scanner for the acquisition of reference data showed that digital impressions by IOSs showed superior trueness compared to open-tray silicone impression in both edentulous and dentate models. A larger impression range tends to deteriorate the trueness of digital impressions.

### Linear and angle errors by laboratory scanners

#### Studies that evaluated digital impression compared to conventional methods

Papaspyridakos et al. evaluated the trueness of digital impressions using Trios and the conventional impression of completely edentulous mandibles [37]. A reference model of an edentulous mandible with five implants was fabricated. Four conventional models were fabricated through conventional polyether impressions using both splinted and non-splinted techniques for both implant- and abutment-level impressions. The reference model and conventional models were scanned using a dental laboratory scanner as the reference and control data, respectively. The STL data from the digital impression and the four conventional impressions were superimposed with the STL data from the reference model to assess the 3D deviations. The trueness of the digital impression did not differ from the following conventional impressions: splinted implant level, splinted abutment level, and non-splinted abutment level models. On the other hand, the trueness of the non-splinted implant-level impressions was inferior to that of digital impressions. Additionally, an implant angulation of up to 15° did not affect the trueness of the digital impression and conventional impressions (Table 5).

Roig et al. evaluated the trueness of digital impressions using a reference model of a partially edentulous maxilla, which accommodated two parallel implants at #14 and #16 [38]. The reference model was scanned using four IOSs (Omnicam, TDS, TRIOS3, and CS 3600) as test data and a dental laboratory scanner (D810) as the reference data. Three types of conventional impressions (closed tray, open tray non-splinted, and open tray splinted) were created, and the stone models were scanned using a dental laboratory scanner. The STL data acquired by the IOSs and dental laboratory scanners were superimposed using a best-fit algorithm to measure the linear and angle errors between the reference and test data. TRIOS3 and CS3600 showed significantly better trueness than the conventional impression with a closed tray and digital impression with Omnicam and TDS (Table 5).

#### Studies that exclusively evaluated digital impression

Mangano et al. compared the trueness of four IOSs using partially and fully edentulous maxilla models [25]. They used a partially edentulous model with missing teeth #21, 24, 25, and 26, with three implants in #21, 24, and 26. The fully edentulous model had six implants in #16, 14, 11, 21, 24, and 26. Reference data were acquired using a dental laboratory scanner. The trueness evaluations were implemented by superimposing the digital impression data obtained using the IOS with reference data. There were no differences in trueness between the partially and completely edentulous models, whereas significant differences were found between the IOSs (Table 5).

They also conducted the same type of study with five different IOSs using a model with a single missing tooth, a model with a partially edentulous space with multiple missing teeth in a row, and a model with a fully edentulous jaw [24]. The reference models were scanned using five IOSs and a dental laboratory scanner. Unlike the previous study, statistically significant differences were found between the different edentulous types. The different IOSs significantly influenced trueness, as shown in a previous study (Table 5).

## Summary of results from studies that utilised dental laboratory scanner for trueness evaluation

Most studies showed that trueness was affected by the IOS manufacturer. Differences in the extent of edentulous space had a significant effect on trueness in some studies, but not in other studies.

## Precision evaluation

### Precision evaluation by distance and angulation in scanned data

Flügge et al. evaluated the precision of digital impressions using three IOSs (iTero, Trios, and TDS) and a dental laboratory scanner by measuring different inter-implant distances and inter-implant angles [15]. They used two different reference models of the mandible: one had an intermediate edentulous space in the lower left and contained two neighbouring implants in #35 and #36, and the other model represented a Kennedy Class I edentulous mandible, with implants in #37, #36, #34, #45, and #47. These models were scanned by the IOSs and a dental laboratory scanner (D250, 3 shapes). The distance and angle between the respective scan bodies were measured on the STL data using the analysis software. The standard deviation of the repeated distance measurements by TDS tended to increase with longer inter-implant distances, whereas iTero and Trios did not show the same tendencies. On the other hand, the angle measurement did not show any deterioration in precision for longer inter-implant distances in any of the scanners (Table 6).

### Precision evaluation by superimposing repeated scanned data by IOSs

Mangano et al. compared the precision of four IOSs (Trios2, CS 3500, Zfx Intrascan, and Planscan) [25]. Two reference models were prepared, representing a partially edentulous model with three implants and a fully edentulous maxilla with six implants. These reference models were scanned by the four IOSs, and the data acquired by the same scanner were superimposed using a best-fit algorithm to evaluate the precision of each IOS. Trios2 and CS 3500 showed significantly better precision than Zfx Intrascan and Planscan, and Zfx Intrascan was significantly better than Planscan (Table 6).

They also conducted the same comparisons for five IOSs (CS 3600, Trios3, Omnicam, DWIO, and Emerald) in another study that investigated the impressions of single missing teeth models as well as in partially edentulous and fully edentulous models [24]. In the single missing tooth situation, CS3600 had the best precision, followed by Trios3, DWIO, Omnicam, and Emerald. In the partially edentulous model, CS 3600 had the best precision, followed by Trios3, Emerald, DWIO, and Omnicam. For the full arch, Trios3 had the best precision, followed by CS3600, Emerald, Omnicam, and DWIO. Significant differences in precision were found between the IOSs and the magnitude of missing teeth (Table 6).

Imburgia et al. compared the precision of four IOSs (CS3600, Trios3, Omnicam, and TDS) in a partially edentulous model with three implants and a fully edentulous model with six implants [27]. The reference models were scanned by each IOS, and the data acquired by the same scanner were superimposed using a best-fit algorithm to evaluate precision. In both the partially and fully edentulous models, they found no statistically significant differences among the different IOSs. For CS 3600, Omnicam, and TDS, the values obtained from the partially edentulous model were significantly better than those obtained from the fully edentulous model. However, no significant differences were found for Trios3 (Table 6).

Miyoshi et al. evaluated the effect of the scanning range on precision [41]. A reference model of an edentulous maxilla with six implants was scanned using four IOSs and a dental laboratory scanner. Conventional silicone impressions were also made, and the stone models were scanned using a dental laboratory scanner. Nine scanning ranges were defined based on the length and number of implants included. In each scanning range, impressions were obtained using each impression method. The data from the repeated scans were superimposed on each other using a best-fit algorithm, and the discrepancies were evaluated. The enlargement of the scanning range deteriorated the precision of the IOSs and conventional impressions. In comparison, the precision of the dental laboratory scanner remained stable irrespective of the size of the scanning range. They concluded that digital impressions by IOSs may show clinically acceptable precision as long as the scanning range is limited, such as within a 3-unit superstructure supported by two implants (Table 6).

Roig et al. evaluated the precision of digital impressions using a reference model of a partially edentulous maxilla, accommodating two parallel implants at #14 and #16 [38]. The reference models were scanned using four IOSs (Omnicam, TDS, TRIOS3, and CS 3600) as test data. Three types of conventional impressions (closed tray, open tray non-splinted, and open tray splinted) were created, and the stone models were scanned with a dental laboratory scanner. The STL data from each repeated measurement for each technique were superimposed using a best-fit algorithm to measure the linear and angle errors between the scans. Digital impressions showed significantly better precision than conventional impression methods (Table 6).

### Summary of the results of the precision evaluation

Similar to the studies that evaluated trueness, the majority of the studies that evaluated the precision of digital impressions showed deterioration of precision as the inter-implant distance or scanning range expanded (Tables 7 and 8). The scanner manufacturer affected the precision of the digital impression. In comparison with conventional impressions, the precision of the digital impression showed comparable or superior results.

**Table 7:** Summary of the comparison of DI and CI, and each parameters’ effect regarding trueness of DI. *CI*, conventional impression; *DI*, digital impression; IOS, intraoral scanner

	DI vs CI	Difference in IOS	Inter-implant distance	Implant angulation	Implant depth	Scan range/edentulous type	Experience of operator
Van der Meer et al. 2012 [13]			△				
Giménez B et al. 2015 [18]				×	×		○
Papaspyridakos et al. 2015 [37]				× (< 15°)			
Ajioka et al. 2016 [14]	DI > CI						
Amin et al 2016 [23]	DI > CI	○					
Mangano et al. 2016 [25]		○				×	
Chia et al. 2017 [31]				○			
Fukazawa et al. 2017 [12]		○					
Gimenez et al. 2017 [40]	DI is acceptable			×			○
Imburgia et al. 2017 [28]		○				○	
Alikhasi et al. 2018 [19]	DI > CI			×			
Marghalani et al. 2018 [9]		○					
Menini et al. 2018 [39]	DI > CI						
Arcuri et al. 2019 [26]				○			×
Di Fore et al. 2019		○	Depends on IOS				
Kim RJY et al. 2019 [28]							
Mangano et al. 2019 [24]		○				○	
Tan et al. 2019 [10]	Depends on IOS		○				

○: significant effect was observed, △: effect was observed without statistical significance, ×: no effect was observed

**Table 8:** Summary of the comparison of DI and CI, and each parameters’ effect regarding precision of DI

	DI vs CI	Difference in IOS	Inter-implant distance	Scan range/edentulous type	Experience of operator
Flügge et al. 2016 [15]			○		
Mangano et al. 2016 [25]		○		×	
Imburgia et al. 2017 [27]		×		Depends on IOS	
Mangano et al. 2019 [24]		○		×	
Miyoshi et al. 2019 [41]		○		○	
Roig et al. 2020 [38]	DI > CI				

○: significant effect was observed, △: effect was observed without statistical significance, ×: no effect was observed

## Effects of clinical parameters on trueness and precision

### Effects of manufacturers

Although several articles compared different kinds of IOSs in terms of trueness and precision, the results are inconsistent among studies. Therefore, the available evidence does not provide decisive data regarding the type of IOS with the best trueness or precision [9, 12, 23–25, 27, 41, 42].

### Effects of the orientation of implants on the accuracy of the digital impression

#### Inter-implant distance

Studies that examined the effects of inter-implant distance on the accuracy of digital impressions consistently suggest that a shorter inter-implant distance allows for better accuracy [15, 42]. Some articles specifically recommend that the indications of digital impressions should be limited to short-span cases, such as 3-unit fixed partial dentures.

On the other hand, the precision of many IOSs did not always deteriorate with longer inter-implant distances [15, 24, 25].

#### Angulation of the implants

Digital impressions of mesially or distally tilted implants have been well documented [9, 16, 18, 26, 28, 29, 40, 43, 44]. One study reported that the angulation of the implant seems to have no detrimental effect on the digital impression accuracy by IOSs [31]. Another study reported high trueness with angulated implants in terms of distance and angle evaluation [43]. On the other hand, conventional impressions of angulated implants have been reported to compromise trueness and precision, probably because the impressions might be deformed when removed [31].

### Effects of scan range

Although some studies report consistent digital impression accuracy irrespective of the scan range [25], the majority of the studies report a gradual distortion of digital impression accuracy as the scan range expands [24, 27, 41]. This can be attributed to the accumulative error of the stitching process. Digital impressions covering large spans are inevitably associated with a larger amount of stitching, thereby making the scan procedure more prone to errors.

### Effects from operators

Two studies evaluated the effects of the operator’s IOS experience on the accuracy of the scanned images [26, 40]. One study that evaluated trueness reported significant effects from the experience of the operators, while the other study that studied precision did not. Since the number of studies is limited, no conclusions can be drawn from the currently available literature.

## Discussion

There has been much debate about the amount of inaccuracy that is acceptable for implant-supported prostheses. Generally, implant-supported prostheses require higher levels of accuracy than tooth-supported prostheses [45]. Therefore, clinicians and dental technicians must strive to make the prosthesis as accurate as possible.

However, it has been reported that some degree of inaccuracy does not cause prosthetic or biological problems. The range of error that does not cause clinical problems is called “biological tolerance” [46] In animal experiments, prosthetic inaccuracy is compensated by the migration of osseointegrated implants to adapt to the prosthesis, which is called “bone adaptation” [47, 48].

Some researchers have proposed a threshold for acceptable error. Andriessen et al. evaluated the accuracy of implants supporting bar attachments for overdentures [20]. They assumed that the threshold of acceptable linear error between two implants was 100 μm and that of the angle error was 0.2°. These are based on the 50-μm lateral movements of the implants when loaded. Therefore, the distance error between the two implants can be up to 100 μm (Kim 2005). The 0.2° angle error threshold is due to the fact that when the tip of a 15-mm implant used in this study was displaced by 50 μm, it tilted by 0.194°. Gintaute et al. adopted 100 μm as the linear error of the inter-implant distance and 0.5° as the angle error without any evidence or references [29].

To evaluate linear errors, the following two methods were used to evaluate linear errors in digital impressions. The first method compares particular inter-implant distances in the reference and test data. The difference in the corresponding inter-implant distance was reported as a linear error. The second method compares the three-dimensional scan body position for the reference and test data.

In cases where two implants are used as abutments, the inter-implant distance is more critical for the fit of the prosthesis than the three-dimensional deviation because the prosthesis is rotated in order to minimise the error. Therefore, research evaluating inter-implant distances is useful. However, in studies with more than three implants, clinicians should refer to the data that incorporates three positional deviations, such as data with *XYZ* coordinates or cumulative 3D deviations that are aligned by best-fit algorithms.

Superimposing the test data on the reference data using a best-fit algorithm makes the error between the data as small as possible. Therefore, the actual deviation of the test data is converted. Guth et al. attempted to solve this problem by placing a straight metal bar on the reference model and used it as a reference point for the superposition in an in vitro study, where they scanned a full arch of natural teeth [35]. Using this method, they found that the deviation of the first quadrant is smaller than that of the second quadrant, which cannot be detected by superimposition using a best-fit algorithm. As proposed in this article, it is possible to evaluate the actual deviation of the digital impression with an object or fixed reference point instead of a best-fit algorithm.

When the implant position is defined by the *XYZ* coordinates, the three-dimensional distance is calculated by the root sum square (RSS = √(*x*^2^ + *y*^2^ + *z*^2^)) in most studies. However, in some studies, other parameters such as the root mean square (RMS = in some cases, the sum of (|*x*|+|*y*|+|*z*|) of the absolute values of √ ((*x*^2^ + *y*^2^ + *z*^2^)/3)) and *XYZ* were calculated and compared. Therefore, readers should be aware of the parameters used when referring to data from digital impression errors.

Another method is to measure the three-dimensional distance directly with software, instead of dropping it into the coordinate axes, as described above. Van der Meer et al. argued that measuring using the *XYZ* coordinate system causes inaccuracies in measurements. Impression accuracy errors often result in very small values. Therefore, the results may change significantly owing to slight deviations of the coordinate axes. However, no study has validated measurements made using software alone, without using the coordinate system.

## Conclusion

Heterogeneity in the research methodology is prevalent among the studies considered here. Therefore, we cannot make a decisive statement regarding the trueness and precision of digital implant impressions by IOSs. So far, the comparison of the numerical values of error between the studies has yet to elucidate any clear answers, despite small methodological differences.

Definitions of the terms relating to impression accuracy as well as the development of a standardised methodology for measurement accuracy that includes validation should be established in order to gather evidence regarding digital impression accuracy.

## Data Availability

All data generated or referenced in this review are included in this manuscript.

## Abbreviations

TDS: True Definition Scanner
TDS2: The second generation of the True Definition Scanner
TDS3: The third generation of the True Definition Scanner
COS: Lava TM Chairside Oral Scanner C.O.S.
